# Maximizing Muscle Hypertrophy: A Systematic Review of Advanced Resistance Training Techniques and Methods

**DOI:** 10.3390/ijerph16244897

**Published:** 2019-12-04

**Authors:** Michal Krzysztofik, Michal Wilk, Grzegorz Wojdała, Artur Gołaś

**Affiliations:** Institute of Sport Sciences, Jerzy Kukuczka Academy of Physical Education in Katowice, ul. Mikolowska 72a, 40-065 Katowice, Poland; m.wilk@awf.katowice.pl (M.W.); wojdala.grzegorz@gmail.com (G.W.); a.golas@awf.katowice.pl (A.G.)

**Keywords:** muscle growth, drop sets, supersets, accentuated eccentric work, blood flow restriction, pre-exhaustion, sarcoplasma stimulating training, movement tempo

## Abstract

Background: Effective hypertrophy-oriented resistance training (RT) should comprise a combination of mechanical tension and metabolic stress. Regarding training variables, the most effective values are widely described in the literature. However, there is still a lack of consensus regarding the efficiency of advanced RT techniques and methods in comparison to traditional approaches. Methods: MEDLINE and SPORTDiscus databases were searched from 1996 to September 2019 for all studies investigating the effects of advanced RT techniques and methods on muscle hypertrophy and training variables. Thirty articles met the inclusion criteria and were consequently included for the quality assessment and data extraction. Results: Concerning the time-efficiency of training, the use of agonist–antagonist, upper–lower body supersets, drop and cluster sets, sarcoplasma stimulating training, employment of fast, but controlled duration of eccentric contractions (~2s), and high-load RT supplemented with low-load RT under blood flow restriction may provide an additional stimulus and an advantage to traditional training protocols. With regard to the higher degree of mechanical tension, the use of accentuated eccentric loading in RT should be considered. Implementation of drop sets, sarcoplasma stimulating training, low-load RT in conjunction with low-load RT under blood flow restriction could provide time-efficient solutions to increased metabolic stress. Conclusions: Due to insufficient evidence, it is difficult to provide specific guidelines for volume, intensity of effort, and frequency of previously mentioned RT techniques and methods. However, well-trained athletes may integrate advanced RT techniques and methods into their routines as an additional stimulus to break through plateaus and to prevent training monotony.

## 1. Introduction

Resistance training (RT) is a primary exercise intervention used to develop strength and stimulate muscle hypertrophy. Increases in muscle mass constitute key components of conditioning in various sports due to the correlation between muscle cross-sectional area and muscle strength [1,2]. Additionally, an increase in muscle mass is one of the goals of bodybuilding [3], and many recreationally strength-trained individuals. Furthermore, adequate levels of muscle mass are an important issue from a health standpoint because its low levels are associated with increased risks of several diseases such as cardiovascular disease [4] and cardio-metabolic risk in adolescents [5] as well as type II diabetes in middle aged and older adults [6].

Muscle hypertrophy occurs when muscle protein synthesis exceeds muscle protein breakdown and results in positive net protein balance in cumulative periods [7]. This could be achieved with both RT and protein ingestion, which stimulates muscle protein synthesis and leads to decreases in muscle protein breakdown [8]. From the nutrition point of view, protein intake alongside RT is a potent stimulus for muscle protein synthesis. With regard to RT, manipulation of its variables such as intensity and volume of effort, exercise order, number of performed repetitions and sets, tempo of movement, and the duration of rest periods between sets and exercises and training status have been extensively explored and discussed to maximize muscle adaptations [9,10]. Volume and intensity of effort are basic components with a direct impact on muscular adaptations [11,12]. The American College of Sports Medicine (ACSM) recommends 1−3 sets per exercise of 8−12 repetitions with 70−85% of one repetition maximum (1RM) for novice and 3−6 sets of 1−12 repetitions with 70−100% 1RM for advanced individuals [13]. However, the recent literature shows a much wider range of training options. Several studies have found that training with low-loads (30−60% 1RM) results in similar hypertrophy to training with moderate and high-loads (>60% 1RM) when volitional fatigue occurs [11,14,15,16]. Moreover, reaching volitional fatigue at all times is not necessary to make significant gains in hypertrophy [17], especially when training with high-loads is considered [18]. Evidence indicates that significant muscle growth occurs when the majority of training sets are performed with ~3–4 repetitions in reserve (with moderate to high-loads) [19]. Furthermore, it has been established that the volume of RT, defined as the total number of repetitions (repetitions x sets), together with loads used for a given exercise, is the key element of adaptation in terms of muscle hypertrophy; moreover, it has been suggested that higher volumes of effort are warranted for maximizing muscle growth response in diverse populations [12,20,21,22,23]. However, following years of training, it becomes difficult to induce further muscle hypertrophy [24], therefore individuals seek advanced resistance training techniques.

The purpose of the present paper was to provide an objective and critical review related to advanced RT methods and techniques influencing skeletal muscle, which may contribute to maximizing muscle hypertrophy in both recreational and competitive athletes.

## 2. Methods

### 2.1. Literature Search

MEDLINE and SPORTDiscus databases were searched from 1996 to September 2019 for all studies investigating the effects of advanced resistance training techniques and methods on muscle hypertrophy and training variables. The search was performed using the following keyword combinations: (‘strength training’ OR ‘resistance training’ OR ‘hypertrophy training’ OR ‘muscle’) AND (‘time under tension’ OR ‘movement velocity’ OR ‘eccentric overload’ OR ‘accentuated eccentric’ OR ‘blood flow restriction’ OR ‘blood flow restricted’ OR occlusion OR ‘cluster set’ OR ‘superset OR ‘agonist-antagonist’ OR ‘pre-exhaustion’ OR ‘drop set’ OR ‘sarcoplasma’ OR ‘advanced training techniques’ OR ‘cross-sectional area’ OR ‘eccentric duration’). The present review includes studies that (1) presented original research data on healthy adult participants in an age range of 19−44 years old; (2) were published in peer-reviewed journals; and (3) were published in the English language. No sex restrictions were imposed during the search stage.

### 2.2. Inclusion and Exclusion Criteria

Research studies investigating the effects of advanced resistance training techniques and methods on muscle hypertrophy and training variables were the primary focus of the literature search. Early screening of the articles was based on titles and abstracts. A total of 1088 studies were initially identified for further scrutiny.

**Figure 1:**
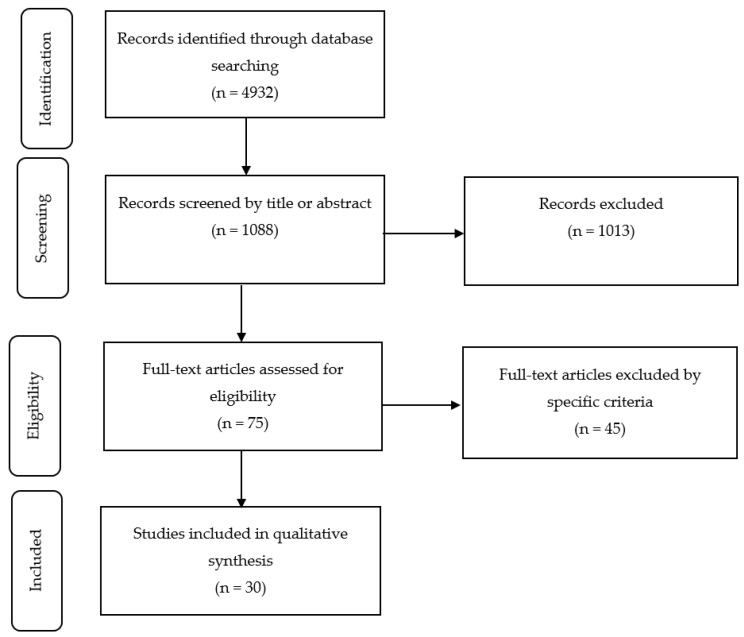
The different phases of the search and study selection.

The next step was to select studies with respect to their internal validity: (1) comparison of different advanced RT techniques and methods with the RT programs performed in traditional training protocols, (2) muscle hypertrophy and/or muscle strength and/or training volume were assessed pre- and post-intervention; for muscle hypertrophy both muscle cross-sectional area changes (magnetic resonance imaging, dual-energy x-ray absorptiometry) and changes in muscle thickness (ultrasound imaging) were considered, while for muscle strength, tests with a repetition maximum (RM) component (e.g., % 1RM or 5RM) were considered; for training volume changes in the number of repetitions, total load and time under tension to muscular failure were considered. The researchers conducted the literature review independently based on inclusion and exclusion criteria. In total, 30 studies met the inclusion criteria for the review (Figure 1).

### 2.3. Results

Experimental details of the studies included in the review (Table 1).

## 3. Discussion

### 3.1. Training Considerations 

Three major factors are emphasized in the conventional hypertrophy model: mechanical tension, metabolic stress, and muscle damage [55]. These factors can occur by optimal manipulation of RT variables and through a wide range of RT techniques. Progressive mechanical tension overload is considered one of the major factors of muscle growth and changes in muscle architecture, which are attained by increasing RT intensity of effort. RT with high-loads (>85% 1RM), and a low number of repetitions (1−5) as well as long rest intervals (~3−5 min) is largely oriented toward a greater magnitude of mechanical tension, which primarily develops strength, while muscle hypertrophy is compromised [13]. RT with a lower number of repetitions, yet with high-loads emphasizes mechanical tension and results in high levels of neural recruitment (fast-twitch muscle fibers). Another critical variable influencing hypertrophy with an evidenced dose-response relationship is RT volume [11,56]. Higher RT volume (28−30 sets/muscle/week) is associated with greater increases in hypertrophy compared to lower volume (6−10 sets/muscle/week) in both untrained and trained populations [12,20]. Implementation of training with moderate number of repetitions (~6−12), multiple sets (3−6), moderate loads (60−80% 1RM), and short rest intervals (60 s) between sets elicits greater metabolic stress (in contrast with high-loads), which appears to be a potent stimulus for inducing muscle hypertrophy [57]. However, as long as RT is performed to volitional fatigue, training load might not affect exercise-induced muscle growth. Findings by Schoenfeld et al. [11] indicate that both low-load RT (≤60% 1RM) performed to volitional fatigue and moderate-load RT (>60% 1RM) elicit significant increases in muscle hypertrophy among well-trained young men. However, the participants following the low-load RT protocol performed approximately three times the total training volume compared to the high-load group (sets × repetitions). Similar findings were also demonstrated in a study by Ikezoe et al. [58], which highlighted the importance of performing exercise to volitional fatigue when low-loads were used to maximize muscle hypertrophy outcomes. These authors compared increments in muscle thickness (rectus femoris) after eight weeks of training with low-load, higher volume (30% 1RM, 12 sets x 8 repetitions) to training with high-load, lower volume (80% 1RM, 3 sets x 8 repetitions) leg extensions in young men. Considering that the training volume in the high-load group was significantly lower than that in the low-load, the degree of muscle thickness attained after intervention was almost twice as high in the high-load group [58]. However, it should be noted that if RT is not conducted to volitional fatigue, reaching the minimum RT intensity threshold (>60%1RM) is necessary to maximize muscle hypertrophy [59].

Furthermore, implementation of advanced RT techniques could provide an additional stimulus to break through plateaus for trained subjects [24] and prevent excessive monotony in training. The most recent RT techniques and methods frequently used by practitioners and coaches include accentuated eccentric loading, prolonged eccentric tempo, cluster sets, high-load RT combined with low-load RT under blood flow restriction, supersets, drop sets, pre-exhaustion, and sarcoplasma stimulating training.

### 3.2. Tempo Eccentric Technique

One of the advanced RT techniques is based on a prolonged duration of the eccentric phase of the movement. The duration of each repetition can be identified by movement tempo, which is determined by four digits (e.g., 2/0/1/0) corresponding to the duration (in seconds) of particular phases of movement (eccentric, transition, concentric, transition) [30]. Changes in the movement tempo during RT impacts the maximal number of repetitions performed in a set, the maximal time under tension, and the final exercise volume [25,26,27]. Several studies have indicated that the use of a faster movement tempo (e.g., 2/0/2/0) results in a significant increase in the maximal number of performed repetitions when compared to the slower tempo (e.g., 6/0/2/0) [25,26,27]. In contrast, a slower tempo of movement, especially during the eccentric phase (e.g., 6/0/2/0), decreases the number of performed repetitions, but extends the time under tension, which may contribute to greater muscle hypertrophy [28]. On the other hand, a meta-analysis of Schoenfeld et al. [60] indicates that similar hypertrophic responses occur when the duration of repetitions ranges from 0.5 to 8 s, although it must be noted that they [60] did not control the duration of particular phases of movement (eccentric vs. concentric), thus making it difficult to draw definite conclusions. Furthermore, a study by Shibata et al. [29] showed that the dominant leg thigh cross-sectional area increased in a similar manner following both the slow (4 s) and the fast (2 s) eccentric phase during the back squat exercise performed to volitional fatigue in a group of male soccer players. In light of the greater force capacity of eccentric actions, and the fact that the energy requirements are typically 4-fold smaller than during the concentric contraction of the same load [61], it would seem logical that lower metabolic stress may occur, which could limit the responses to this training technique.

However, studies indicate a wide range of manipulation of the duration of the eccentric phase of movement can be employed if the primary goal of training is to maximize muscle hypertrophy [29,60]. Although, it is not currently clear whether slow tempo provides a superior stimulus for muscle hypertrophy, from a practical point of view, employing a fast but controlled duration of the eccentric phase (~2s) may allow for a high time-efficiency of training and prevent the excessive time of training sessions.

### 3.3. Accentuated Eccentric Loading Method

Another useful method that can be used during RT, based on eccentric contractions includes accentuated eccentric loading (AEL). This training strategy is based on the muscles’ ability to generate greater force during maximal eccentric (~20−60%) versus other types of contraction. The use of weight releasers allow for overloading the muscles during the eccentric phase of movement due to its specific construction. The weight can be unloaded in the transition from the eccentric to the concentric phase of movement. The use of high-loads during the eccentric phase of movement is associated with significant exercise induced muscle damage and mechanical tension, which have been associated with a hypertrophic response [55]. Furthermore, some studies have shown that performing eccentric-only contractions led to higher gains in muscle mass when compared to concentric-only actions [30,62]. Nonetheless, recent literature has indicated that when the volume of training was matched, both AEL and high-load RT led to similar hypertrophic responses in groups of strength-trained athletes [31,32,63]. Furthermore, RT protocols that did not promote significant muscle damage still induced similar muscle hypertrophy in comparison with those protocols that promoted initial muscle damage [7]. However, differences appear in muscle architecture adaptations. Training with the concentric-only phase led to muscle growth mainly by the addition of sarcomeres in parallel (increased pennation angle with little change in fascicle length), while training with eccentric-only contractions led by the addition of sarcomeres in series (increased fascicle length and a small increase in the pennation angle) [64]. 

Furthermore, due to the greater mechanical tension, it could provide an additional hypertrophic stimulus [31,33,65]. Although it must be noted that the main disadvantage of this technique is the necessity of weight releasers or the presence of experienced spotters during training. Moreover AEL, requires the eccentric load to reload after every repetition, thus is possible that the inter-repetition rest may excessively extend the time of particular repetitions and the whole training session.

### 3.4. Low-Load Resistance Training Under Blood Flow Restriction 

Another RT method that allows for the avoidance of high mechanical stress associated with high-load RT and the high training volumes required when exercising with low-loads to volitional fatigue is to combine RT under blood flow restriction (BFR) [34,66,67]. The BFR method involves the application of a restrictive device (a tourniquet, an inflatable cuff, or elastic wraps) on the proximal part of the limb to reduce the arterial blood flow and to occlude the venous return [67]. Such an intervention results in an accumulation of metabolic products distal to the restriction and when coupled with RT, drastically increases metabolic stress. However, with regard to low-load RT under BFR, a significant increase in the muscle cross-sectional area was observed even without reaching volitional fatigue in particular sets [35]. Furthermore, several studies have suggested that increases in muscle mass following low-load RT under BFR (20−30% 1RM) do not exceed those observed after the use of high-load RT (80% 1RM) without BFR [36,37,38]. The effectiveness of using BFR concerns various populations such as non-athletes [39,40], moderately experienced participants (>1 year) [36], and elite athletes [41,42]. High-load RT with additional low-load sets under BFR may elicit beneficial muscular responses in healthy athletes [68]. 

The most frequently and evidence-based repetition and set scheme involves 30 repetitions in the first set followed by three sets of 15 repetitions with 30 s rests in between with 20−40% 1RM and pressure, which contribute to 40−80% of arterial occlusion pressure [69]. However, it must be noted that BFR induced muscle growth is limited to the limb muscles [43].

### 3.5. Cluster Sets Technique

Another RT technique that partly allows for the balance of both mechanical tension and metabolic stress consists of cluster sets. In a traditional scheme of sets, repetitions, a chosen group of exercises are performed consecutively, with a long inter-set rest interval, are then followed by another set of repetitions. On the contrary, cluster sets include short, inter-set rest intervals (20−60s) with a lower number of repetitions [70]. Previous research has mostly investigated the effects of cluster sets on force production, power output, and movement velocity, while findings related to muscle hypertrophy are limited [44]. Nevertheless, implementation of inter-set rest intervals allows for a greater RT volume to be achieved for a particular external load when compared with a traditional scheme of sets [44,45] in trained and untrained men, possibly providing an additional stimulus for muscle hypertrophy. However, it should be noted that cluster sets induce less metabolic stress, but greater emphasis is placed on mechanical stress due to the use of higher training intensities of effort in comparison with traditional sets [44,45,46,71]. Thus, the implementation of cluster sets with short inter-set rest intervals could be a useful strategy to carry out high-volume sessions of high-loads, while keeping a high time-efficiency of training (training volume/time). Furthermore, cluster sets may serve as an alternative to traditional sets for promoting muscle hypertrophy over time during parallel periodization models [46], and prevent monotony in training. Moreover, future studies should investigate the direct effects of cluster sets on exercise-induced muscle growth. 

### 3.6. Supersets and Pre-exhaustion Technique

Supersets and pre-exhaustion during RT can be defined as a pair of different exercise sets performed without rest. Supersets most commonly consist of two exercises for the same muscle group [47], agonist-antagonist muscles [48,72] or alternating upper and lower body muscle groups [49] consecutively followed by a recovery period; pre-exhaustion involves performing a single-joint before a multi-joint exercise for the same muscle group (e.g., dumbbell fly before the bench press). In a study by Wallace et al. [47], supersets (flat bench press followed by the incline bench press) resulted in a significantly lower volume of training than a traditional exercise order in strength-trained males. However, with regard to agonist–antagonist supersets, investigation by Robbins et al. [48] (bench pull paired with the bench press) indicated a significantly higher training volume when compared to a traditional exercise order. Furthermore, this type of superset as well as upper–lower body supersets were found to be more time-efficient than traditional exercise order sessions [48,49].

The pre-exhaustion technique is commonly used by bodybuilders seeking to enhance the muscle growth of target muscles. The rationale for this technique is that performing a single-joint exercise first fatigues the agonist in isolation, thereby placing greater stress on the agonist and increasing its activation during multi-joint exercise and potentiating its hypertrophy [73]. Another variation is the reverse pre-exhaustion (e.g., triceps pushdown before the bench press), and the justification for this approach is that the fatigued synergist contributes less to the subsequent multi-joint exercise, thereby placing greater stress on the agonist group [74]. However, a study by Golas et al. [75] partially disagreed with this statement as the results indicated that a pre-exhaustion exercise (incline dumbbell fly) did not affect the pectoralis major activity during the flat bench press exercise at 95% 1RM. Despite that, pre-exhaustion of the synergist muscles (triceps brachii and anterior deltoid before the bench press) led to their higher activation during the multi-joint movement (bench press) as compared to the baseline [75]. Furthermore, results of a study by Soares et al. [50] suggested that pre-exhaustion (triceps pushdown followed by the bench press) decreased the maximal number of repetitions performed during a set to volitional fatigue. 

In conclusion, practitioners aiming to maximize training volume and intensity of effort may be well advised to consider the use of supersets (agonist–antagonist and upper–lower body) in their RT programs. The use of these exercise orders may be more time-efficient than the traditional approach, and especially useful when time limitations exist in the planning of training sessions.

### 3.7. Drop Sets and Sarcoplasma Stimulating Training Technique

Drop sets involve performing a set to volitional fatigue with a given load and then immediately reducing the load (e.g., ~20%) and continuing the exercise until subsequent volitional fatigue [76]. Briefly, the rationale for this technique is high metabolic stress induced due to a high number of repetitions performed with short rest intervals. Accordingly, a study by Fink et al. [51] showed significantly higher muscle thickness after drop sets in comparison with RT following a traditional sets scheme, which can be considered as a potential marker for metabolic stress [57]. Furthermore, results of the study by Fink et al. [51] showed significant increases in the triceps cross-sectional area after six weeks of drop sets training when compared to traditional sets. Nevertheless, it must be noted that participants taking part in this research were recreational trained persons with little experience in RT (did not regularly train for more than one year). On the other hand, Angleri et al. [52] demonstrated that drop sets did not promote greater lower body muscle growth when compared with traditional sets in well-trained males when training volume was equalized.

Similarly to drop sets, sarcoplasm stimulating training (SST) consists of sets of exercises performed at 70–80% 1RM to volitional fatigue and then repeating this protocol twice more with 20 s rest intervals in between. The next step is to reduce the external load by 20% and perform an additional set with a 4/0/1/0 tempo; following a 20 s rest interval, 20% of the external load is reduced again, and a set with 4/0/1/0 tempo is completed to volitional fatigue. In the last set, the load is further decreased by 20% and after its completion, following a 20 s rest interval, a static hold is performed (e.g., at 90° of elbow flexion) to volitional fatigue [53]. Another SST variation refers to the performance of eight sets of exercises at 70−80% 1RM to volitional fatigue with programmed rest intervals between subsequent sets (45, 30, 15, 5, 5, 15, 30, and 45 s) without reducing the load [53]. Similarly, to drop sets, the main aim of SST is to induce high metabolic stress [53]. Recently, de Almeida et al. [53] demonstrated that SST resulted in greater acute biceps brachii and triceps brachii muscle thickness when compared to a traditional set scheme in trained subjects, even when total training volume was higher in a traditional set scheme RT.

Evidence suggests the beneficial effects of both drop sets and SST in acute increases in triceps brachii muscle thickness [53] in both amateur and well-trained subjects, even with lower training volume versus a traditional set scheme RT. However, studies that have investigated the chronic effects of drop sets did not show a superior hypertrophy response when compared with traditional sets [52,54]. Moreover, the chronic effects of SST on muscle growth have not been examined yet. 

### 3.8. Limitations

The present review has several limitations that should be addressed. The majority of included studies did not control nutritional intake, which can affect the magnitude of muscle adaptations. Another limitation relates to studies that examined the influence of advanced methods and techniques on training variables, but did not analyze hypertrophic responses and/or strength improvements, which would be the basis for explaining their efficiency. In addition, only one study [44] directly compared the responses between trained and untrained participants.

## 4. Conclusions

Considering the aforementioned studies, effective hypertrophy-oriented training should comprise a combination of mechanical tension and metabolic stress. In summary, foundations for individuals seeking to maximize muscle growth should be hypertrophy-oriented RT consisting of multiple sets (3−6) of six to 12 repetitions with short rest intervals (60 s) and moderate intensity of effort (60−80% 1RM) with subsequent increases in training volume (12–28 sets/muscle/week) [20]. Moreover, trained athletes may consider integrating advanced resistance training techniques and methods to provide an additional stimulus to break through plateaus, prevent monotony, and reduce the time of training sessions. Evidence suggests some beneficial effects for selected RT techniques especially in the case of training volume, time-efficiency, and intensity of effort. Furthermore, even though most of these techniques and methods did not show a superior hypertrophy response compared to the traditional approach, it may serve as an alternative to prevent monotony or it could improve readiness to training sessions. To maintain high time-efficiency of training and when time limitations exist, the use of agonist–antagonist, upper–lower body supersets, drop sets, SST, and cluster sets may provide an advantage to the traditional approach. Furthermore, the employment of fast but controlled tempo (~2 s) and supplementation of high-load RT with low-load RT under BFR may allow for high time-efficiency of training and prevent excessively long training sessions. With regard to the higher degree of mechanical tension, the use of AEL in RT should be considered, therefore, in cases where time is limited, cluster sets might be a better choice. The implementation of drop sets, SST, and low-load RT under BFR could provide time-efficient techniques to increase metabolic stress. In summary, currently there is not sufficient evidence to provide specific guidelines for volume, intensity of effort, and frequency of the previously mentioned resistance training techniques.

Furthermore, persistence in training and diet is essential. Recently, research has shown that muscle hypertrophy that occurs at initial stages of RT (~4 sessions) is mostly attributable to muscle damage induced cell swelling with the majority of strength gains resulting from neural adaptations (8−12 sessions). Within the latter phase of RT (6−10 weeks), muscle growth begins to become the dominant factor [7].

## Figures and Tables

**Table 1:** Experimental details of the studies included in the review.

Reference	Sample	Training Method/Technique	Training Duration	Exercise Prescription	Conditions	Were Repetitions Performed to Volitional Fatigue?	Measurement Variables	Conclusions
Wilk et al., 2018 [25]	42 trained males	Tempo ECC	Acute	Bench Press	2/0/2/0 vs. 5/0/3/0 vs. 6/0/4/0	Yes	TVOL	Regular movement tempo resulted in highest REP to failure but with the lowest total TUT.
Hatfield et al., 2006 [26]	9 trained males	Tempo ECC	Acute	Back Squat and Shoulder Press	10/0/10/0 vs. volitional movement tempo	Yes	TVOL	Volitional movement tempo resulted in higher REP to failure.
Sakamoto and Sinclair 2006 [27]	13 males	Tempo ECC	Acute	Bench Press	slow vs, medium vs. fast vs. ballistic	Yes	TVOL	Fast movement velocity resulted in the highest REP to failure.
Burd et al., 2012 [28]	8 males	Tempo ECC	Acute	Knee Extension	6/0/6/0 vs. 1/0/1/0	Yes	TVOL	Slow movement tempo resulted in higher TUT.
Shibata et al., 2018 [29]	24 male soccer players	Tempo ECC	6 weeks	Parallel Back Squat	4/0/2/0 vs. 2/0/2/0	Yes	HT, STH	Both protocols lead to significant increase in muscle HT, but longer ECC duration was less effective in STH improvement.
English et al., 2014 [30]	40 males	AEL	8 weeks	Leg Press and Calf Press	0, 33, 66, 100, or 138% of 1RM	No	HT, STH	AEL lead to the highest increases in muscle HT and STH.
Brandenburg and Docherty 2002 [31]	18 males	AEL	9 weeks	Preacher Curls, Supine Elbow Extensions	75% vs. 120% 1RM	Yes	HT, STH	AEL lead to higher increase in STH for supine elbow extension, with no significant changes in muscle HT in both groups.
Walker et al., 2016 [32]	28 trained males	AEL	10 weeks	Leg Press and Unilateral Knee Extension	6RM Leg Press, 10RM Unilateral Knee extensions vs. 140% 1RM	Yes	HT, TVOL	AEL lead to higher increase in work capacity (REP to failure), but not muscle HT.
Friedmann-Bette et al., 2010 [33]	25 trained males	AEL	6 weeks	Unilateral Knee Extensions	8RM vs. 1.9-fold higher for ECC	Yes	HT, STH	Both protocols lead to significant increase in muscle HT and STH.
Loenneke et al., 2012 [34]	20 (10 males and 10 females) trained	BFR	Acute	Bilateral Knee Extension	30% 1RM BFR vs. 30% 1RM without BFR	Yes	TVOL	BFR reduced REP to failure.
Kubo et al., 2006 [35]	9 males	BFR	12 weeks	Unilateral Knee Extensions	20% 1RM BFR vs. 80% 1RM without BFR	No	HT	Both protocols lead to significant increase in muscle HT.
Lowery et al., 2014 [36]	20 males	BFR	4 weeks	Biceps Curls	30% 1RM BFR vs. 60% 1RM without BFR	No	HT	Both protocols lead to significant increase in muscle HT.
Farup et al., 2015 [37]	10 males	BFR	6 weeks	Dumbbell Curls	40% 1RM BFR vs. 40% 1RM without BFR	Yes	HT, TVOL	Both protocols lead to significant increase in muscle HT, with reduced REP to failure in BFR.
Ellefsen et al., 2015 [38]	9 untrained females	BFR	12 weeks	Unilateral Knee Extensions	30% 1RM BFR vs. 6−10RM without BFR	Yes	HT	Both protocols lead to significant increase in muscle HT.
Laurentino et al., 2012 [39]	29 males	BFR	8 weeks	Bilateral Knee Extension	20% 1RM without BFR vs. 20%1RM BFR vs. 80%1RM without BFR	No	HT, STH	BFR lead to increase in muscle HT and STH with the same degree as high-intensity RT.
Lixandrao et al., 2015 [40]	26 males	BFR	12 weeks	Bilateral Knee Extension	20 or 40% 1RM + BFR (40 or 80%AOP) vs. 80% 1RM without BFR	No	HT, STH	When BFR protocols are performed at very low intensities higher AOP is required. BFR protocols significantly improved muscle HT and STH, but with less effect in STH.
Yamanaka et al., 2012 [41]	32 athletes	BFR	4 weeks	Bench Press and Back Squat	20% 1RM BFR vs. 20% 1RM	No	HT, STH	BFR significantly improved muscle HT and STH.
Cook et al., 2018 [42]	18 males	BFR	6 weeks	Leg Press and Knee Extension	70% 1RM vs. 20% 1RM BFR	Yes (only last set)	HT, STH	Both protocols significantly improved muscle HT and STH, but BFR was less effective.
Yasuda et al., 2011 [43]	30 males	BFR	6 weeks	Bench Press	75% 1RM vs. 30% 1RM BFR	No	HT, STH	Both protocols significantly improved muscle HT and STH, but BFR was less effective.
Oliver et al., 2015 [44]	23 (12 trained and 11 untrained) males	CS	Acute	Back Squat	4 sets of 10 REP vs. 4 sets of 2 CS of 5 REP at 70% 1RM	No	TVOL	CS allowed to lift a greater TVOL load with reduced TUT.
Iglesias-Soler et al., 2014 [45]	9 athletes	CS	Acute	Parallel Back Squat	3 sets to muscular failure of TS or CS	Yes	TVOL	CS lead to higher REP to failure.
Tufano et al., 2017 [46]	12 trained males	CS	Acute	Back Squat	3 sets of 12 REP vs. 3 sets of 3 CS of 4 REP vs. 3 sets of 6 CS of 2 REP at 60% 1RM	No	TVOL	CS protocols lead for greater external loads and higher TUT.
Wallace et al., 2019 [47]	11 trained males	SS/Pre-Exhaustion	Acute	Bench Press, Incline Bench Press, Triceps Pushdowns,	TS vs. SS (agonists) vs. pre-exhaustion (single-joint + multi-joint exercise) vs. pre-exhaustion (multi-joint + single-joint)	Yes	TVOL	SS (agonists) decreased TVOL load.
Robbins et al., 2010 [48]	16 trained males	SS/Pre-Exhaustion	Acute	Bench Press, Bench Pull	SS vs. TS	Yes	TVOL	SS (agonist-antagonist) increased total TVOL load.
Weakley et al., 2017 [49]	14 trained males	SS/Pre-Exhaustion	Acute	Back Squat, Bench Press, Romanian Deadlift, Dumbbell Shoulder Press, Bent Over Row, Upright Row	TS vs. SS vs. tri-sets	No	TVOL	SS (upper-lower body, agonist-antagonist) and tri-sets protocols were more efficient (kilograms lifted per minute) than TS.
Soares et al., 2016 [50]	14 trained males	SS/Pre-Exhaustion	Acute	Bench Press and Triceps Pushdowns	pre-exhaustion vs. TS	Yes	TVOL	Total TVOL load lifted is reduced when multi-joint exercise is performed after single-joint.
Fink et al., 2018 [51]	16 males	DS/SST	6 weeks	Triceps Pushdowns	3 sets of TS vs. single DS	Yes	HT	Single set of DS lead to higher muscle HT.
Angleri et al., 2017 [52]	32 males	DS/SST	12 weeks	Leg Press and Knee Extension	DS vs. TS vs. crescent pyramid	Yes	HT, STH	All protocols significantly improved muscle HT and ST.
de Almeida et al., 2019 [53]	12 trained males	DS/SST	Acute	Biceps Curls and Triceps Pulley Extensions	TS vs. SST	Yes	HT, TVOL	SST lead to greater acute muscle HT, with reduced training time, even with a lower total TVOL load.
Ozaki et al., 2018 [54]	9 untrained males	DS/SST	8 weeks	Dumbbell Curls	3 sets of 80%1RM vs. 3 sets of 30%1RM vs. 1 set of 80%1RM and then four DS at 65%, 50%, 40% and 30%1RM	Yes	HT, STH, TVOL	A single high-load set with additional four DS increased muscle HT and STH as well as work capacity (REP to failure), with an reduced training time.

ECC: eccentric; TVOL: training volume; HT: hypertrophy; STH: strength; REP: repetitions; TUT: time under tension; AEL: accentuated eccentric loading; 1RM: one-repetition maximum; ECC: eccentric; BFR: blood flow restriction; RT: resistance training; AOP: arterial occlusion pressure; CS: cluster set; TS: traditional set; SS: superset; DS: drop sets; SST: sarcoplasma stimulating training.
